# Special Issue on Machine Learning and AI for Sensors

**DOI:** 10.3390/s23052770

**Published:** 2023-03-03

**Authors:** Panagiotis Pintelas, Sotiris Kotsiantis, Ioannis E. Livieris

**Affiliations:** Department of Mathematics, University of Patras, 265-00 Patras, Greece

This article summarizes the works published under the “*Machine Learning and AI for Sensors*” (https://www.mdpi.com/journal/sensors/topical_collections/ML_AI_sensors accessed on 14 February 2023) Issue of the *Sensors* journal. The primary aim of this Special Issue is to demonstrate the recent advances related to machine learning and AI methods on sensors as well as investigate the impact of their application in a variety of hard real-world problems. In total, twelve (12) research manuscripts were accepted for publication after going through a careful peer-review process based on contribution and quality criteria. All accepted manuscripts possess significant elements of novelty and enclose several application domains, which provide the readers with a glimpse of the state-of-the-art research in the machine learning area.

In recent decades, new advances in machine learning (ML) and artificial intelligence (AI) have covered areas from zero- and single-shot algorithms [1,2,3] to deep and complex neural network algorithms [4,5] for vast amounts of data. On the other hand, advances in sensing technology (local and remote) are responsible for a profound impact on our daily life and all aspects of current industrial and technological developments. Combining the two areas creates an extremely interesting, challenging and promising interdisciplinary area of research, deploying new forefront research and development opportunities. In more simple words, the integration of ML and AI methods, together with sensors technology and engineering, is able to benefit other areas such as Industry 4.0, Demotic Systems, Internet of Things, etc. Along this line, a variety of intelligent systems based on ML and AI methodologies have been proposed, evaluated and applied, exploiting sensor data in many scientific areas [6,7,8,9,10].

However, the increasing challenging necessities of the industrial sector as well as considerable needs of this data-driven era led to the development of new, efficient and robust methodologies and approaches. Along this line, novel AI and ML algorithms are needed, such us new data quality techniques, new clustering, classification and reinforcement learning methods; in addition, distributed AI algorithms are required and different strategies are needed to embed these algorithms in sensors.

The first paper is entitled “*A novel approach to image recoloring for color vision deficiency*” and it is authored by Tsekouras et al. [11]. In this paper, the authors proposed a novel method to modify color images for the protanopia and deuteranopia color vision deficiencies, which admits color contrast enhancement and preserves image naturalness criteria. In the process, four (4) modules were employed: (i) fuzzy-clustering-based color segmentation for extracting key colors of the input image; (ii) the key colors were mapped onto the CIE 1931 chromaticity diagram, and through an intelligent mechanism, were discriminated by the color blind; (iii) the key colors were further adapted by optimizing a regularized objective function, which combines the aforementioned criteria; (iv) the recolored image was obtained by color transfer, which involves the adapted key colors together with the associated fuzzy clusters. The proposed methodology was compared against three relative state-of-the-art methods over 195 natural images and six digitized art paintings. The comprehensive experimental evaluation and the statistical analysis revealed the superiority of the proposed approach.

The second paper is entitled “*Xcycles backprojection acoustic super-resolution*” and it is authored by Almasri et al. [12]. The authors proposed a new model architecture, named XCycles BackProjection, for the acoustic image super-resolution problem along with the Acoustic Map Imaging VUB-ULB dataset (AMIVU). The proposed XCBP model utilizes the iterative correction procedure in each cycle for reconstructing the residual error correction for the encoded features in both a low- and high-resolution space, while the developed AMIVU dataset provides large simulated and real captured images at different resolutions. The performance evaluation process showed that the proposed model outperformed traditional feedforward state-of-the-art models. Finally, the authors stated that an advantage of their proposed methodology is that it is able to considerably reduce the sub-sampling error produced during data acquisition.

The third paper is entitled “*Decision confidence assessment in multi-class classification*” and it is authored by Bukowski et al. [13]. In this research, the authors proposed a new approach to the assessment of decision confidence when multi-class recognition is concerned. The proposed approach aimed to provide a solution for considerably decreasing the amount of work, which needed to be conducted by human experts while evaluating different samples. In more detail, it focuses on evaluating each considered sample in terms of decision confidence instead of being assigned to a single label/class (hard classification). The advantages of the proposed methodology are that it can be easily adjusted to any number of classes and it is able to focus either on the coverage of the training dataset or on the classification accuracy, based on user preferences. The evaluation process was based on a dataset which contained a total of 8526 of manually labeled images showing holes drilled by steadily declining tools. Based on the presented experimental results, the authors stated that it provides an acceptable quality of approach.

The fourth paper is entitled “*Boosting intelligent data analysis in smart sensors by integrating knowledge and machine learning*” and it is authored by Łuczak et al. [14]. In this work, the authors proposed a hybrid neural architecture which enables intelligent data analysis efficacy to be boosted in smart sensor devices. The proposed architecture is composed by two interacting functional modules arranged in a homogeneous, multiple-layer architecture: a knowledge sub-network module and a conventional neural sub-network module. The former implements knowledge in the conjunctive normal form using a three-layer structure based on new types of learnable units, called *L*-neurons, while the latter is a fully-connected three-layer feedforward neural network. The authors provided a comprehensive experimental analysis, which showed that the proposed hybrid structure successfully combines learning and knowledge and provides high recognition performance even for a small number of training instances. Finally, the authors stated that since the proposed *L*-neurons are able to learn through classical backpropagation processes, the proposed architecture is capable of updating and repairing its knowledge.

The fifth paper is entitled “*Hyperspectral image classification using deep genome graph-based approach*” and is authored by Tinega et al. [15]. The authors proposed a new model for hyperspectral image classification, named GGBN (Deep Genome Graph-Based Network) for tapping the potential of genome graphs and hybrid models. GGBN process spectral-spatial data using a 3D-convolutional layer followed by a series of 2D-convolutional layers for enhancing the accuracy and scalability of hyperspectral image classifications. The proposed model was encapsulated via three (3) well-known classification experiments using Indian Pines (IP), Salinas Scene (SS) and University of Pavia (UP) datasets. In their experimental analysis, the authors reported that GGBN outperformed traditional state-of-the-art models and presented a classification accuracy of 99.97%, 99.74% and 96.85% over IP, SS and UP datasets, respectively, using only 5% of the labeled data for training.

The sixth paper is entitled “*A heterogeneous RISC-V processor for efficient DNN application in smart sensing system*” and is authored by Zhang et al. [16]. In this approach, the authors attempted to considerably improve the computational performance, under the limitation of low power consumption for obtaining a satisfactory energy-efficiency ratio. More specifically, they proposed a lightweight and flexible pipeline-integrated deep neural network learning architecture, which was compatible with open-source RISC-V instructions. In addition, the dataflow of deep neural network is organized by the very long instruction word (VLIW) pipeline, which combines the proposed special intelligent enhanced instructions together with the Single Instruction Multiple Data (SIMD) parallel processing unit. The presented numerical experiments demonstrated that total power consumption is about 411MW while the total power efficiency is approximately 320.7 GOPS/W.

The seventh paper is entitled “*A convolutional autoencoder topology for classification in high-dimensional noisy image datasets*” and is authored by Pintelas et al. [17]. In this research, the authors proposed a new approach for dealing with the problem of deep convolutional neural network models’ vulnerability to noise and redundant information that are usually encapsulated into the high-dimensional raw input images, and are able to lead to unreliable and unstable predictions. More specifically, they proposed a convolutional autoencoder model for filtering out and compressing redundant information and noise from initial high dimensionality input images. Then, the compressed output of the model is fed into a convolutional-based neural network. For experimentally proving the efficiency of their approach, they conducted a series of experiments on three well-known and complex benchmarks, evaluating their methodology against traditional approaches for classifying noisy images. The experimental analysis revealed the robustness and the advantages of the proposed approach.

The eighth paper is entitled “*Multiclass image classification using gans and cnn based on holes drilled in laminated chipboard*” and it is authored by Wieczorek et al. [18]. The authors deal with the problem of reducing the damage caused by a blunt tool and proposed an intelligent model for identifying different levels of quality of the holes. Notice that the reduced quality prediction would serve as a warning that the drill is about to wear down. In their research, they used a real-world dataset along with a data-augmentation technique based on general adversarial networks for obtaining more training instances and handling data imbalance. The proposed model was based on a convolutional neural network, which was hyper-tuned and evaluated against Microsoft’s Custom Vision provided by the Azure platform. Their experimental results displayed the efficiency of their approach and the predicting accuracy of the developed model.

The ninth paper is entitled “*A comprehensive survey on nanophotonic neural networks: architectures, training methods, optimization and activations Functions*” and it is authored by Demertzis et al. [19]. In this work, the authors presented an in-depth overview of the materialization and development methods of neuromorphic circuits of nanophotonic arrangements [20] for every respective contemporary architecture of conventional neural networks as well as the advantages and restrictions which arise during the transition to the optical from the electronic materializations. The main contribution of this research is that it provides an extensive literature review of the most well-known architectures, optimizations, activation functions and training methods of the nanophotonic networks. In addition, it provides a comprehensive meta-review analysis of the advantages and disadvantages of nanophotonic networks.

The tenth paper is entitled “*Multi-agent reinforcement learning via adaptive Kalman temporal difference and successor representation*” and is authored by Salimibeni et al. [21]. The authors proposed the Multi-Agent Adaptive Kalman Temporal Difference (MAK-TD) framework and its Successor Representation-based variant, named MAK-SR. Their primary aim was the development of an alternative for addressing, with sample inefficiency, memory problems and issues of a lack of prior information regarding deep neural network-based multi-agent reinforcement learning techniques. The proposed MAK-TD/SR frameworks consider the continuous nature of the action-space, which is associated with high-dimensional multi-agent environments and with exploiting Kalman Temporal Difference to deal with the parameter uncertainty. Both proposed frameworks were evaluated in a series of experiments, which were implemented through the OpenAI Gym MARL benchmarks. In these experiments, a different number of agents in competitive, cooperative and mixed scenarios were used, which demonstrated that the proposed MAK-TD/SR frameworks exhibit superior performance than their state-of-the-art counterparts.

The eleventh paper is entitled “*An IoT-enabled platform for the assessment of physical and mental activities utilizing augmented reality exergaming*” and it is authored by Koulouris et al. [22]. In this research, the authors presented a prototype platform for exergames, which combined Internet of Things (IoT) and augmented reality (AR) on commodity mobile devices for the development of serious games in the healthcare domain. The primary aim was to promote the use of gamification methodologies to boost the users’ physical activities and to considerably assist the cognitive statuses as well as the regular assessment of their health through quests and challenges in the real and virtual world. In addition, the authors stated that the novelty of the proposed solution was the combination of an integrated and modular environment, together with state-of-the-art technologies and tools for providing benefits to both users and healthcare professionals. The developed platform was able to monitor, in real-time, the users’ biosignals and activities during a game through the integration of wearable devices and sensors for data collecting for each session, of which were analyzed by healthcare professionals. Finally, the proposed solution was validated in a series of real-world scenarios while the provided results from the simulations were analyzed in order to further improve the performance and usability of the prototype.

The twelfth paper is entitled “*A robust artificial intelligence approach with explainability for measurement and verification of energy efficient infrastructure for net zero carbon emissions*” and is authored by Moraliyage et al. [23]. In this work, the authors proposed a comprehensive AI approach for Measurement and Verification (M&V) protocols in energy-efficient infrastructure. The main contribution of their framework lies in its use of all relevant data (pre- and post-energy conservation measures) for the development of efficient and explainable predictive models for energy-saving estimations. The global and local explainability features were provided by the utilization of Shapley Additive exPlanations [24]. The proposed framework was evaluated on a large real-world dataset of energy consumption data within a multi-campus tertiary education institution setting of La Trobe University, which was composed of 200 buildings of diverse operational functions and sensor technologies. The reported experimental results provided empirical evidence regarding the efficiency and validity of the proposed approach for robust and explainable M&V for energy-efficient building infrastructures as well as net-zero carbon emissions.

Conclusively, we point out that the rationale and motivation behind this Special Issue was to provide a minor contribution to the existing literature about machine learning and AI methods for sensors. The examination of a variety of interesting proposed methodologies led to the presentation of a diverse range of novel strategies. Our great expectation is that the presented techniques and approaches, which were demonstrated in this Special Issue, will be found to be constructive and deeply appreciated by the scientific and industrial communities. Finally, the guest editors express their sincere gratitude to all authors for their high-quality contributions as well as the publisher and members of staff for their invaluable advice and support, which contributed in a decisive manner to enriching the quality of this editorial paper. 
